# Vectorial adaptive optics: expanding the frontiers of optical correction

**DOI:** 10.1038/s41377-023-01358-1

**Published:** 2024-01-30

**Authors:** Qiming Zhang, Min Gu

**Affiliations:** Institute of Photonic Chips, University of Shanghai for Science and Technology, Shanghai, 200093 China

**Keywords:** Adaptive optics, Imaging and sensing

## Abstract

Researchers at the University of Oxford have introduced a groundbreaking technique called vectorial adaptive optics (V-AO), which extends the capabilities of traditional adaptive optics to correct for both polarization and phase aberrations. This novel approach opens new possibilities for manipulating the complex vectorial field in optical systems, enabling higher-dimensional feedback correction.

Adaptive optics (AO) has been widely used to correct phase aberrations in various optical systems, ranging from astronomical telescopes to super-resolution microscopes^1–3^. However, the role of polarization aberrations in degrading system performance has often been overlooked. The team at Oxford addresses this limitation by introducing V-AO, which encompasses the correction of both polarization and phase aberrations^4^. The researchers demonstrate the implementation of V-AO using sensor feedback, sensorless AO, or a hybrid approach combining aspects of both. By correcting for common vectorial aberration sources, such as objective lenses and biological samples, they validate the improvements achieved in the vector field state and focal quality of optical systems.

The significance of this research lies in its ability to provide feedback control over extra vectorial degrees of freedom, pushing the boundaries of traditional scalar beam shaping. By manipulating the complex vectorial field, V-AO paves the way for next-generation AO functionality. The applications of V-AO are vast and diverse. In the field of astronomy^5^, V-AO can enhance the detection and analysis of celestial objects, enabling more accurate measurements and observations. In optical microscopy^6^, it can improve the resolution and contrast of images, leading to advancements in biological and medical research. Moreover, V-AO has potential applications in laser-based nanofabrication, especially in anisotropic materials^7–9^, where precise control over the vectorial field is crucial for achieving desired outcomes. It can also find utility in biomedical characterization, aiding in the study of biological samples with higher accuracy and fidelity (Fig. 1).

**Fig. 1:**
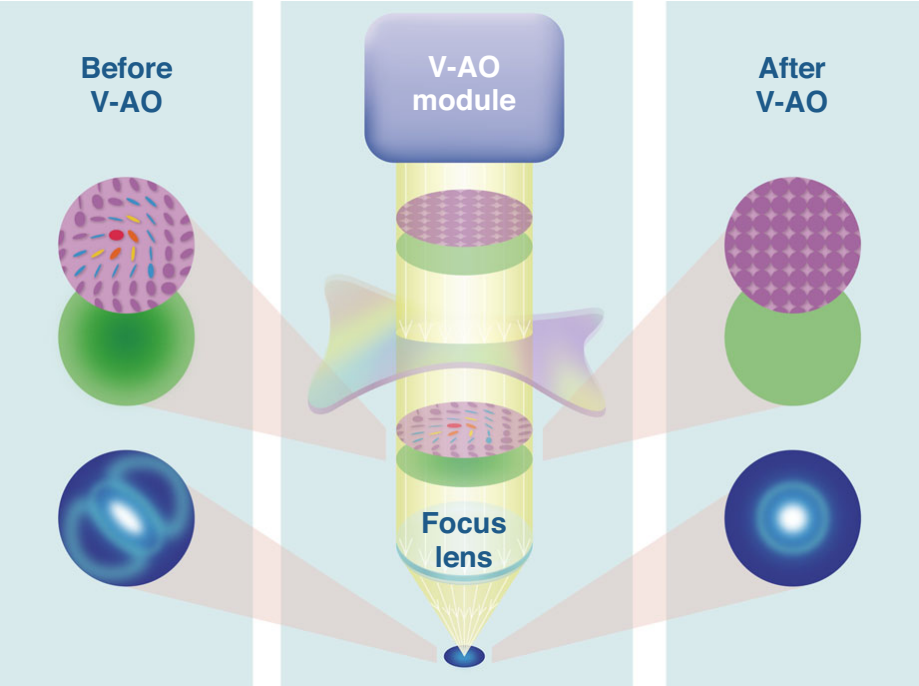
Schematic drawing of vectorial adaptive optics (V-AO)

Particularly, it is worth noting that V-AO is a versatile technique for correcting vectorial aberrations in optical systems for complex optical multiplexing. V-AO can be applied to artificial neural networks enabled by nanophotonics multiplexed in different dimensions^10,11^. V-AO can also be applied to super-resolution and multi-dimensional optical data storage to achieve smaller recording bits and more multiplexing channels toward Petabyte data storage^12^.

The researchers envision further advancements in V-AO by expanding the number of AO devices, allowing for even more comprehensive vectorial aberration compensation. By conjugating the vectorial state in various optical paths, such as the emission path of a microscope, the capabilities of V-AO can be extended.

The introduction of vectorial adaptive optics represents a significant breakthrough in the field of optical correction. By addressing both polarization and phase aberrations, V-AO offers enhanced control over the vectorial field, leading to improved system performance and expanded applications. This research opens up new avenues for exploration and innovation in various scientific and technological domains, promising exciting developments in the future.
